# Not the doctor’s business: Privacy, personal responsibility and data rights in medical settings

**DOI:** 10.1111/bioe.12711

**Published:** 2020-02-14

**Authors:** Carissa Véliz

**Affiliations:** ^1^ University of Oxford Uehiro Centre for Practical Ethics Wellcome Centre for Ethics and Humanities Faculty of Philosophy United Kingdom of Great Britain and Northern Ireland

**Keywords:** confidentiality, data minimization, data rights, egalitarianism, luck, medical ethics codes, personal responsibility, privacy

## Abstract

This paper argues that assessing personal responsibility in healthcare settings for the allocation of medical resources would be too privacy‐invasive to be morally justifiable. In addition to being an inappropriate and moralizing intrusion into the private lives of patients, it would put patients’ sensitive data at risk, making data subjects vulnerable to a variety of privacy‐related harms. Even though we allow privacy‐invasive investigations to take place in legal trials, the justice and healthcare systems are not analogous. The duty of doctors and healthcare professionals is to help patients as best they can—not to judge them. Patients should not be forced into giving up any more personal information than what is strictly necessary to receive an adequate treatment, and their medical data should only be used for appropriate purposes. Medical ethics codes should reflect these data rights. When a doctor asks personal questions that are irrelevant to diagnose or treat a patient, the appropriate response from the patient is: ‘none of your business’.

## 1 INTRODUCTION

Public healthcare systems are under considerable pressure to deliver the best possible treatment to each and every one of their patients. Factors that contribute to strained healthcare systems include ageing populations, the rising prevalence of chronic illnesses, the expense of cutting‐edge technology, and austerity cuts to public health budgets. Faced with scarcity, societies need to find ways to distribute medical resources as fairly as possible, in a way that can be justifiable to those who lose out—the patients who do not receive the best possible treatment, or who, owing to long waiting lists, receive it later than what would have been ideal.

Increasing evidence suggests that individual lifestyle choices such as smoking, physical inactivity, bad eating habits and unsafe sex are among the top risk factors for disease burden.1

*Global health risks. Mortality and burden of disease attributable to selected major risks* (2009). Retrieved from http://www.who.int/healthinfo/global_burden_disease/GlobalHealthRisks_report_full.pdf

 The realization that individuals’ choices can have a significant impact on their health has inspired proposals to distribute medical resources, or costs, according to criteria that take into account personal responsibility. What is most attractive about proposals that factor in personal responsibility is that, in the spirit of luck egalitarianism, they seem to treat all citizens equally and fairly. They distribute resources according to factors (putatively) within the control of those who lose out, rather than according to factors over which people have little or no influence (e.g. race, gender, etc.). Under such a scheme, everyone seems to have an equal chance to receive the best possible treatment available, if only they make the right choices. To those who lose out and receive less than others (or receive it more slowly, or for a higher price), resource allocators have a seemingly adequate justification: 'you could have received the same care if you had made better choices'. Proposals like these might be all the more tempting in the digital age, given how much more data it is possible to collect and analyse.

I will not go through all the arguments that have been proposed in favour and against considering responsibility within healthcare allocation. Kerith Sharkey and Lynn Gillam have mapped out the literature, categorizing the arguments on both sides of the debate.2
Sharkey, K., & Gillam, L. (2010). Should patients with self‐inflicted illness receive lower priority in access to healthcare resources? Mapping out the debate. *Journal of Medical Ethics, 36*, 661–665.10.1136/jme.2009.03210220817816
 They conclude that the debate has stagnated and is in need of new views. This paper puts forward an as‐yet unexplored argument: that assessing patients’ responsibility for their health with the objective of limiting access to healthcare on the basis of that information would be too privacy‐invasive to be morally justifiable. Of course, doctors will often have to ask patients about their habits and behaviour to diagnose and treat them appropriately, and some of those questions can be very sensitive. In this paper, I am concerned only with the collection and use of such sensitive information for the purposes of limiting access to healthcare according to criteria of responsibility (i.e. finding out to what degree a patient is responsible for their illness in order to restrict their access to healthcare). Such a privacy intrusion would put patients at risk, damage the doctor–patient relationship, and would likely not even serve justice. I argue that patients have a right to refrain from sharing with their doctor personal information that is not relevant for their diagnosis and treatment, and that medical ethics codes should include a principle of data minimization.

## 2 RESPONSIBILITY ASSESSMENT AND THE DOCTOR–PATIENT RELATIONSHIP

Two reasons that relate to the doctor–patient relationship can be found in the literature against factoring in personal responsibility in the allocation of healthcare. The first claims that such policies would undermine patients’ trust in healthcare professionals, and even impel patients to lie to their doctors. Leonard Glantz, for instance, argues that denying medical treatment to smokers would make it more likely that patients might lie to their doctors about their smoking.3
Glantz, L. (2007). Should smokers be refused surgery? *British Medical Journal, 334*(7583), 21.10.1136/bmj.39059.532095.68PMC176408117204800
 The second reason maintains that it is inappropriate for healthcare professionals to judge or punish patients, and that doctors should only take into account medical considerations.4
Sharkey & Gillam, op. cit.; Shelley, E. (1996). Coronary artery bypass surgery in smokers. *Heart, 75*(6), 544–545. 10.1136/hrt.75.6.544PMC4843738697153
 On this point, Glantz contends that ‘[w]ithholding surgery from smokers (…) distorts the modern doctor–patient relationship, which is based on partnership’.5
Glantz, op. cit.



These considerations are often expressed more as comments in passing, rather than as detailed arguments. My objective in this paper is to argue that at least part of the reason why patients would lose trust in healthcare professionals, and why it would be inappropriate for doctors to assess the personal responsibility of their patients, is related to privacy issues.

The only mention of privacy I have found in the literature on the ethics of assessing personal responsibility in healthcare settings is by John Harris, and it is a short parenthetical consideration. He writes that, even if it were possible to collect all the relevant information that would be necessary to assess responsibility, ‘there remains the question of whether it would be desirable for other reasons (which would include privacy and the dangers of abuse)’.6
Harris, J. (1995). Could we hold people responsible for their own adverse health? *Journal of Contemporary Health Law and Policy, 12*, 147–153.8907109



Given that confidentiality is one of doctors’ fundamental duties towards their patients, it seems that privacy concerns are particularly important in medical contexts, and their neglect is a conspicuous mistake.

## 3 MEDICAL CONFIDENTIALITY AND PRIVACY

Past and present medical ethics codes usually recognize duties of confidentiality—that is, duties of non‐disclosure of information shared in the context of a fiduciary, contractual or professional relationship such as that of the doctor and patient. Confidentiality is a tool to protect patients’ privacy. Concerns about medical privacy go as far back as the Hippocratic Oath, which included a vow not to speak of what is seen and heard in the course of treatment.7
Oath of Hippocrates. (1995). In W. Reich (Ed.), *Encyclopedia of bioethics* (p. 2632). New York, NY: Macmillan.
 The prominence of confidentiality in ethics codes signals the importance of privacy in medical settings, but in order to fully respect and protect privacy, it is not enough to refrain from disclosing information about patients. As I will argue, it is also important to minimize the collection of information, and to use sensitive information only for appropriate purposes (which, in the medical context, is treatment).

One loses informational privacy when others access personal information about oneself. Personal information is the kind of information we have good reason to keep to ourselves, or to share only with a few trustworthy others. It is the kind of information that can make one vulnerable to embarrassment, discrimination and other types of harm such as identity theft.

Privacy is valuable for both intrinsic and instrumental reasons. Peeping Toms make us uncomfortable—even when they are not a threat in any way. Instrumentally, privacy is valuable insofar as it contributes to other desirable goals, such as physical and financial security. If people do not know where you live or work, it is harder for them to physically stalk you. If people do not know your full name and credit card number, it is harder for them to steal your money. Privacy protects us from other harms such as discrimination, public shame and reputational damage. It contributes to autonomy by giving us enough physical and mental space to be ourselves and to develop our views without undue external influence. In short, privacy protects us from the burdens and risks of social interaction, and in so doing fosters certain worthwhile pursuits.

Medical privacy is a particularly important kind of privacy. Disease not only leaves us vulnerable to worry, pain, deterioration of the body, and possibly even death—it also leaves us vulnerable to social harms such as stigma, discrimination, shame and exploitation. The information that someone needs medical care is to social predators what the smell of blood is to sharks.

Patients can face many harms as a result of medical data breaches. If a prospective employer has information on a job applicant suggesting some medical concerns, they might be tempted to discriminate against her and hire someone else. Such discrimination would be very hard to prove, as the victim might have no reason to suspect that she is a victim. Insurance companies could take advantage of medically relevant information, such as genetic tendencies, to charge some people more than others. Pharmaceutical companies could engage in price discrimination by identifying people who desperately need a medicine that can be bought only from them, and charge them more for it. Hackers could commit identity theft. Criminals could extort patients, threatening to expose sensitive images or information about them. In 2017, for instance, a criminal group accessed sensitive data from a Lithuanian cosmetic surgery clinic and extorted patients, asking for a bitcoin ransom. Hackers then published more than 25,000 private photos, including nude ones, and personal data that included passport scans and national insurance numbers.8
Hern, A. (2017, May 31). Hackers publish private photos from cosmetic surgery clinic. *The Guardian* Retrieved from https://www.theguardian.com/technology/2017/may/31/hackers-publish-private-photos-cosmetic-surgery-clinic-bitcoin-ransom-payments




As these examples show, collecting and storing sensitive data with devices connected to the internet is riskier than when records were kept on paper. Personal data is sensitive, hard to safeguard, and coveted by many—insurance companies, banks, prospective employers, hackers and criminals, governments and intelligence agencies, among others. In an economy that is more and more dependent on data, personal information is valuable. But it is also vulnerable, which in turn makes patients and any institution storing sensitive data vulnerable as well. A data breach could lead to many disasters for the institution responsible for the data—from loss of reputation to a lawsuit, potentially costing a hospital a fortune. In cyberspace, attackers have an advantage over defenders. While the attacker can choose the moment and method of attack, the defender has to protect against every kind of attack at all times.9
Schneier, B. (2016). Data is a toxic asset, so why not throw it out? *CNN* Retrieved from https://edition.cnn.com/2016/03/01/opinions/data-is-a-toxic-asset-opinion-schneier/index.html




Data breaches are so common in medical settings that it is unrealistic to suppose that the safety of patients’ data can be guaranteed. In 2015, over 112 million health records were breached in the United States alone.10
Munro, D. (2015, Dec 31). Data breaches in healthcare totaled over 112 million records in 2015. *Forbes* Retrieved from https://www.forbes.com/sites/danmunro/2015/12/31/data-breaches-in-healthcare-total-over-112-million-records-in-2015/ ‐ 5118fabc7b07
 While the number of patients affected was lower in 2017, the number of healthcare data security incidents was higher than in previous years, and seems to be on the rise, suggesting that patients’ health records are increasingly at risk.11

*Largest Healthcare Data Breaches of 2017* (2018). Retrieved from https://www.hipaajournal.com/largest-healthcare-data-breaches-2017/

 In 2019, ProPublica reported that the medical records of more than 5 million patients in the United States and millions more around the world are unprotected on the internet.12
Gillum, J., Kao, J., & Larson, J. (2019, Sep 17). Millions of Americans' medical images and data are available on the internet. Anyone can take a peek. *ProPublica* https://www.propublica.org/article/millions-of-americans-medical-images-and-data-are-available-onthe-internet

 The best way to protect patients and medical institutions from privacy disasters is to collect and store as little sensitive data as possible.

## 4 THE CASE FOR DATA MINIMIZATION AND APPROPRIATE USES OF DATA

A defence of data minimization in medical settings starts with noting the sensitivity of medical data, the risk involved in collecting and storing data, and the devastating consequences that a data breach can bring about.13
Data minimization is a requirement of the new European General Data Protection Regulation (enforceable as of 25 May 2018).
 A further weighty element to take into consideration in medical settings is patient vulnerability. Patients in the doctor’s office and in hospitals are typically not at their best. Stripped of makeup, fancy clothes, and other social veils, they are often feeling unwell, worried about their condition, and at the mercy of medical professionals to provide them with the care they need. Their negotiation capacities are by and large limited by their circumstances. If protecting one’s privacy is difficult on a good day and under ideal conditions, it is even harder to do under challenging circumstances. Given this extreme situation of vulnerability on the part of patients, it is the duty of healthcare professionals to minimize privacy losses, show consideration towards patients, and avoid any unnecessary exposure.

Consider going to the doctor’s office on account of a stomach pain. After asking all the relevant questions—where does it hurt, do you feel any nausea, etc.—the doctor starts collecting information that does not seem directly relevant to your health issue. She might ask about your sexual preferences, your shopping habits, or the make and model of your car. When you ask for an explanation regarding these tangential questions, she responds that the hospital requires that information in order to sell it to insurance companies. We would clearly consider such behaviour an unjustified intrusion into the patient’s privacy. The example is slightly exaggerated, just to show that there seem to be implicit norms and expectations as to the kind of data that doctors should collect and the purposes that they should use the data for. But the example is not as outlandish as one would hope: in 2014, *The Telegraph* reported that the U.K.’s National Health Service (NHS) sold 13 years of hospital data covering 47 million patients to insurance companies.14
Donnelly, L. (2014, Feb 23). Hospital records of all NHS patients sold to insurers. *The Telegraph* https://www.telegraph.co.uk/news/health/news/10656893/Hospital-records-of-all-NHS-patients-soldto- insurers.html




The doctor is in a privileged position with respect to the patient: she has personal access to him in a position of authority. Meanwhile, the patient is in a vulnerable position. He is likely feeling unwell and scared about his health, and he needs the doctor to access adequate treatment. To use that position of authority for anything other than to help the patient seems unjustifiable.

Yet the principle that doctors do not collect more information than what is strictly necessary is nowhere to be seen in medical ethics codes. The World Medical Association’s International Code of Medical Ethics, for example, recognizes the right to confidentiality, but does not mention the importance of minimizing privacy intrusions. In contrast, the American Medical Association states that physicians should ‘minimize intrusion on privacy’, but does not specify what that entails. In the U.K., the Caldicott Principles recommend that healthcare professionals  ‘use the minimum necessary personal confidential data’, but it is unclear what is meant by ‘use’. The Principles do not mention the collection of data, only using and sharing it. It is also unclear what is meant by ‘minimum necessary’. I contend that ethical codes should recognize a duty not to collect sensitive information that is not necessary for diagnosis and treatment. A data minimization principle should recommend that healthcare professionals limit personal data collection, storage and usage to data that is necessary for diagnosing and treating patients (as well as for medical research, in medical research settings). Patients should also be allowed to ask questions and make consultations that can remain off the record at their request (as long as there is no risk to other people).

Ethical codes that do not mention or are not explicit enough on the importance of data minimization for the protection of privacy are out of date. They need to be updated, first, because they do not take into account the risks of data collection in the digital age, and, second, because such neglect amounts to a remnant of more authoritarian times in medicine, when it was up to doctors what to ask and do, and patients had less of a say in managing their risks and health. Giving up personal information can constitute a serious privacy loss, as well as a risk, and patients should be free to keep to themselves information that is not necessary for obtaining adequate treatment.

It might not always be easy to determine what is medically necessary information and what is not. Sometimes the job of a physician can resemble that of a detective. On occasion, the answers to questions that might seem irrelevant might contain the key to the puzzle of what is making a patient sick, and past behaviour can be a major part of assessing a patient’s medical condition. Physicians could be at risk of poor clinical care if they did not gather enough medical data.

As long as questions are made with the objective of healing the patient, data collection is justified. Relevant questions are those that will help the doctor to diagnose and treat. There are two ways in which the patient’s right to privacy could be violated: if the doctor collected more data than she would if she only had diagnosis and treatment for that patient in mind, or if the data collected was used for purposes other than the diagnosis and treatment of the patient without his or her consent.

Helen Nissenbaum’s framework of contextual integrity helps to explain the importance of using medical data for the treatment and diagnosis of that data subject.15
Nissenbaum, H. (2010). *Privacy in context. Technology, policy, and the integrity of social life*. Stanford, CA: Stanford University Press.
 Ensuring that personal information flows *appropriately* is just as important as data minimization. Context is what determines that appropriateness, and in the doctor’s office, what is appropriate is to collect and use data for the purposes of diagnosis and treatment, as well as for medical research, if patients have given their consent for such use. Along with a principle of data minimization, then, medical codes should include a principle establishing appropriate uses of data. Medical data should be used for medical purposes, with few justifiable exceptions.16
Medical purposes may not be the only appropriate and justifiable use of medical data. On occasion, medical data might need to be shared with the police in the course of a criminal investigation, for example. Such exceptions are rare, and not commercial in nature.
 It should not be sold to third parties, and it should not be used to assess patients’ responsibility.

## 5 ASSESSING RESPONSIBILITY IS PRIVACY‐INVASIVE AND RISKY

One might think that patients’ privacy is already at risk, given that, in order to provide them with adequate treatment, sensitive medical information will necessarily be collected and stored. It is reasonable to ask what, if anything, would change if personal responsibility were to be taken into account.17
I am grateful to an anonymous reviewer for this objection.
 Privacy risks to patients would increase significantly if personal responsibility were to be given consideration within healthcare because significantly more data would be gathered on them—and, in particular, some of the data (e.g. on habits) would be particularly attractive to insurance companies, data‐brokers and hackers, among others. The greater the amount of data that is collected, the more accurate the responsibility assessment, and the greater the privacy risks.

If doctors had in mind the goal of investigating responsibility, it is very likely that this would lead them to ask more questions than they would otherwise. Patients would need to be asked about their sexual practices and partners, eating habits, alcohol consumption and drug use, visits to the gym, hygiene practices, work, level of stress, and social network, as social isolation and bad relationships are among the many health risk factors over which individuals have some degree of control.18
House, J. S., Landis, K. R., & Umberson, D. (1988). Social relationships and health. *Science, 241*(4865), 540–545.10.1126/science.33998893399889
 In his critique of luck egalitarianism, Jonathan Wolff argues that people are humiliated when they are forced to reveal things about themselves that they find shameful. According to him, ‘in a society of equals no one would be prepared to carry out, or submit to, such inspections, even if they were required by justice’.19
Wolff, J. (1998). Fairness, respect, and the egalitarian ethos. *Philosophy and Public Affairs, 27*(2), 97–122.



Consider the case of someone who was once an alcoholic and now needs a liver transplant. As Colin E. Atterbury points out, if we were to assess the personal responsibility of drinkers, we would need to determine how much they drank, whether they knew that amount to be excessive, whether they drank out of habit or addiction, their genetic predisposition to addiction (with genetic data being some of the most sensitive data that can be gathered about someone), what their social network was like, and more.20
Atterbury, C. E. (1996). Anubis and the feather of truth: Judging transplant candidates who engage in self‐damaging behavior. *Journal of Clinical Ethics, 7*(3), 268–276.8981198
 It would not be necessary to collect any of that information merely to treat a patient who needs a liver transplant. Yet every extra data point collected puts patients’ privacy at greater risk. Even if such sensitive information were to be anonymized, the more data points we have on individuals, the easier it is to identify them.21
Weber, G. M., Mandl, K. D., & Kohane, I. S. (2014). Finding the missing link for big biomedical data. *JAMA, 311*(24), 2479–2480. 10.1001/jama.2014.4228 24854141
 In some cases, only two or three data points are necessary to identify someone.22
de Montjoye, Y. A., Hidalgo, C. A., Verleysen, M., & Blondel, V. D. (2013). Unique in the crowd: The privacy bounds of human mobility. *Sci. Rep., 3*, 1376; de Montjoye, Y. A., Radaelli, L., Singh, V. K., & Pentland, A. S. (2015). Identity and privacy. Unique in the shopping mall: On the reidentifiability of credit card metadata. *Science, 347*(6221), 536–539.10.1126/science.125629725635097



Given that patients may lie about their habits or engage in self‐deception, it might be necessary to corroborate their word with other sources of information. It would be helpful to gain access to data collected by social media, files held by data‐brokers, and data from wearables such as digital watches. Such research would be expensive and time‐consuming. When scarcity is one of the main justifications for introducing personal responsibility as a criterion for allocating medical resources, spending valuable resources carrying out medically unnecessary and invasive research on patients rather than on curing them seems unpalatable.

The more detailed people’s dossiers are, the more profitable they are, which, in the current data economy, makes it more likely that they will be stolen or sold. Detailed information about people’s genetic tendencies and habits could be very valuable. Crossing the boundaries of moral limits, data‐brokers have been known to sell lists of rape victims, alcoholics, HIV patients, and erectile dysfunction sufferers.23
Hill, K. (2013, Dec 19). Data broker was selling lists of rape victims, alcoholics, and 'erectile dysfunction sufferers'. *Forbes* Retrieved from https://www.forbes.com/sites/kashmirhill/2013/12/19/data-broker-was-selling-lists-of-rape-alcoholism-and-erectile-dysfunction-sufferers/ ‐ 761e73d21d53. For more on the trade of medical data, see Tanner, A. (2017). *Our bodies, our data. How companies make billions selling our medical records*. Boston, MA: Beacon Press.
 Data about personal responsibility can reveal much about individuals’ characters, habits and relationships. From gambling websites to payday loan websites, there are innumerable businesses and other agents lustful to learn about people’s vulnerabilities and weaknesses of will. The best way the healthcare profession can protect their patients’ medical privacy is to collect the bare minimum information that is needed to treat them—nothing more.

## 6 THE LEGAL TRIAL OBJECTION

While critics might grant that assessing personal responsibility in healthcare settings would be invasive and constitute a data risk for patients, they might still think that those downsides are necessary to achieve justice. On this view, it is seen as unfair that people who take care of themselves may be assigned lower medical priority on account of factors outside their control, while people who act irresponsibly with their health may be assigned the same or higher priority. Furthermore, people who do not make much use of the healthcare system on account of their healthy habits may feel that, through their taxes, they are paying for others’ recklessness. In order to achieve justice, the critic might argue, a proper investigation is necessary, just like we allow for such investigations in the context of a legal trial in the justice system.

However, if we allow suspected criminals the privilege against self‐incrimination, it would be unfair not to allow that right to patients. There is something perverse in forcing a person to do something that goes against her own interest. While the right against self‐incrimination might rule out forcing patients to confess to bad habits, an independent investigation into patients’ lifestyles might still be in order, just as investigations are carried out in legal trials.

The justice system and the healthcare system are not analogous, however. In a legal trial, someone has been accused of breaking the law. If the defendant denies being guilty, an investigation must ensue to ascertain who is wrong or lying—the defence or the prosecution—and who is owed what. The investigation is part and parcel of treating citizens as equals—the prosecutor’s word is given the same weight as the defendant’s word, as both have to prove their case. Judges and juries are impartial parties that assess the relevant evidence and make a decision. Judges and juries owe their loyalty to neither prosecutors nor defendants—only to justice.

In contrast, for the doctor–patient relationship to be one of trust and cooperation, healthcare professionals owe their loyalty to their patients—not to the system. The assessment of responsibility in healthcare settings would introduce a kind of conflict of loyalty for healthcare professionals. The job of healthcare professionals is to be on the side of patients, doing what they can to improve their patients’ health. If they had to judge the responsibility of patients, they would be forced to ask questions knowing that the patient’s answers may have a negative effect on his health by positioning him lower on the waiting list, for example. The business of the doctor is to heal, not to judge, and in order to be good at her job, a doctor has to be her patients’ advocate. Here I am advancing a view of the doctor–patient relationship akin to ‘the healing relationship’ articulated by Edmund Pellegrino, according to which the common goal of healing is the essence of the medical endeavour.24
Pellegrino, E. D. (2006). Toward a reconstruction of medical morality. *American Journal of Bioethics, 6*(2), 65–71.10.1080/1526516050050860116500860
 To transform doctors into judges or gatekeepers to resource allocation would be to betray the healing relationship.

The interaction between patients and doctors can have significant health effects. If the patient feels positively about his doctor, the interaction may produce placebo effects, thereby helping the patient improve his health. If the patient feels negatively about his interaction with healthcare professionals—if he feels judged, or that his privacy is being violated—those feelings might induce nocebo effects that have a negative impact on his health.25
Benedetti, F. (2013). Placebo and the new physiology of the doctor–patient relationship. *Physiol. Rev., 93*(3), 1207–1246.10.1152/physrev.00043.2012PMC396254923899563



It could be argued that responsibility could be assessed without implicating doctors. A specialized external medical team could do it, for instance, or a hospital manager. But patients would likely still feel distrust towards the healthcare system, even if their doctors were not directly involved in assessing their responsibility. They might also consider doctors as accomplices of a harsh system. If patients perceive the healthcare system as a judgmental and merciless one, they are unlikely to think any better of healthcare professionals working for such a system.

People’s integrity and trustworthiness is partly appraised on the basis of the organizations they work for—think of Nazi officers (as an extreme case), Facebook employers (it was once ‘cool’ to say that one worked for the tech company; not anymore, after the various privacy scandals), or, as a positive example, physicians working for *Doctors Without Borders*. When an organization implements a policy thought to be unethical, people expect ethical employers to resign or rebel; otherwise, they are likely to be considered accomplices. When, in 2012, the Spanish government passed a law to exclude illegal immigrants from the healthcare system, more than 1,500 doctors refused to comply, making a public pledge that they would offer medical care to anyone who needed it. In their campaign, doctors appealed to their ‘right to cure’.26
Sevillano, E. G., García de Blas, E., & Rico Motos, C. (2012, Aug 26). Crearemos una red que atienda a todos. *El País* Retrieved from https://elpais.com/politica/2012/08/26/actualidad/1346001950_940230.html

 Healthcare professionals who value their patients’ medical needs above any other consideration are more likely to be perceived as benevolent and trustworthy by such patients.

Furthermore, who carries out the invasive research is irrelevant from the point of view of privacy. When citizens go to court, they are expecting to receive a just outcome. Invasive research into people’s private lives is often necessary to find out the relevant facts that are in turn necessary for juries and judges to serve justice. The privacy invasion is justified because it is necessary in order to attain what citizens want when they go to court: justice. In contrast, when citizens go to the hospital, they want healing. Any privacy invasion that is unnecessary to fulfil that purpose seems unjustified—particularly given that privacy invasions put people in danger.

Moreover, the proposal that someone other than doctors judge patients’ responsibility does not avoid the criticism of intrusion made to luck egalitarianism. Elizabeth Anderson has argued that assessing people’s responsibility ‘makes demeaning and intrusive judgments of people’s capacities to exercise responsibility and effectively dictates to them the appropriate uses of their freedom’.27
Anderson, E. S. (1999). What is the point of equality? *Ethics, 109*(2), 287–337.
 Personal responsibility criteria may not be as impartial as they seem at first glance, as they are not neutral with respect to different lifestyles, often falling prey to moralizing social biases.28
Friesen, P. (2018). Personal responsibility within health policy: Unethical and ineffective. *Journal of Medical Ethics, 44*, 53–58; Wilkinson, S. (1999). Smokers' rights to health care: Why the 'restoration argument' is a moralising wolf in a liberal sheep's clothing. *Journal of Applied Philosophy, 16*(3), 255–269.10.1111/1468-5930.0012815497230
 We value some risky lifestyles and stigmatize others for reasons having nothing to do with justice. For example, we tend to look more benevolently upon people who freely choose to live in a city with dangerous levels of air pollution than upon alcoholics. In liberal democracies, unless a crime has been committed, there is no legitimate authority to judge a person’s values and life choices except that person herself.

A final consideration related to the legal trial objection has to do with proportionality and punishment. In criminal systems, the death penalty either does not exist at all, or is reserved for the very gravest crimes. Yet denying someone medical assistance or assigning them lower priority could mean sentencing them to death. Even if one believed that the healthcare system is an appropriate place to determine matters of justice, surely death or serious injury is a disproportionate punishment for not taking better care of oneself. Illness seems like punishment enough. Besides, if we punished the sick, we would only be punishing the unlucky ones, as other people engaging in equally risky practices (e.g. drinking exactly the same amount of alcohol) do not get sick. What makes the difference between a minority of drinkers who develop cirrhosis and a majority who do not is partly luck (in the form of genetic susceptibility to injury from alcohol).29
Atterbury, op. cit.



## 7 CONCLUSION

This paper has argued that assessing personal responsibility in healthcare settings for the allocation of medical resources would be too privacy‐invasive to be morally justifiable. In addition to being an inappropriate intrusion into the private lives of patients, it would put patients’ sensitive data at risk, making data subjects vulnerable to a variety of privacy‐related harms. Even though we allow privacy‐invasive investigations to take place in legal trials, the justice and healthcare systems are not analogous. The duty of doctors and healthcare professionals is to help patients as best they can—not to judge them. If we allow suspected criminals the privilege against self‐incrimination, surely we should not force patients to give information that will be used against their best interest. Patients should not be forced into revealing any more personal information than what is strictly necessary to receive an adequate treatment, and medical data should only be used for appropriate purposes. Medical ethics codes should reflect these data rights. In medical settings, when you are asked personal questions that are irrelevant to your diagnosis or treatment, an appropriate response is: ‘none of your business’.

## CONFLICT OF INTEREST

The author declares no conflict of interest.

